# An Epoch in Medical History

**Published:** 1892-12

**Authors:** 


### AN EPOCH IN MEDICAL HISTORY.

The meeting of representatives of Southern Medical Colleges, which took place at Louisville, Kentucky, on the 16th November, ult., an account of which is published herewith, had been looked forward to with much anxiety, as so much for the future weal or woe of medicine depended upon its results. Unanimity of action on one point, at least, was indispensable,—the lengthening of the lecture term from two to three courses, and a higher standard of requirement for the degree. Unless all agree to this reform, it would be impracticable.

A painful recollection of the utter failure of a similar attempt, was in the minds of many, and hence the anxiety to know if all the Colleges would agree upon an advance, and stick to it. The JOURNAL'S recollection is, that an agreement of this kind was entered into once before, by at least a majority of the reputable schools; but when a certain distinguished College found a big falling off in its matriculates the next year, the flag was hauled down, and a return was promptly made to the former status of two courses and easy conditions. Our recollection is that this recreant was Bellevue. This one occurrence demonstrated how difficult is the task of securing good faith on the part of the Colleges in carrying out a reform which so nearly touches the pocket of the teachers; and it demonstrates, also, what the JOURNAL has always insisted upon as the only proper course,—that all medical teachers should be independent of the revenue of the school, and the licensing power taken from them. They should be paid by the State, or by other means than the fees from students.

The meeting was fairly representative, though not as largely attended as was hoped for. It will be seen, by reference to the *Rules and Regulations of the Association*, elsewhere published, that an agreement was entered into to require three courses of lectures, of six months each, as a condition to the degree; but the standard of requirement for matriculation is too low; that is, there is a loop hole of escape which will, we fear, prove fatal to the real object and intent of the movement;—the provision that if the applicant have not "a diploma from some literary or scientific institution of learning," or a "certificate from some legally constituted high school," or "general superintendent of State education," he may be admitted on a certificate of the "superintendent of some *country board* of public education, attesting the fact that he is possessed of at least the educational attainments required of second grade teachers of public schools." And if he fail in even this slim requirement, he may be admitted to lectures anyhow, on the *promise to study up*,—"qualify himself in the required literary departments."

That amounts to practically no requirement for matriculation. The idea of an ignorant man, one who cannot attain the grade of second class teacher in a country school, "qualifying himself in the literary departments" while he is attending medical lectures, is absurd. A student at lectures, who means business, is compelled to give his whole time—day, and half of the night—to medical study, clinics, dissections, laboratory work, etc.; and many of them, the first course, to *spelling* out the hard Latin and Greek names in medical nomenclature, and looking up their meaning in the dictionary. What time (to say nothing of inclination) would such a student have to "qualify himself in literary departments"?

This very low requirement is in direct opposition to the declared object and purpose of the organization,—to "elevate the standard of medical education by *requiring a more thorough preliminary training*," etc.; it is tantamount to requiring nothing, in the way of literary education.

Necessarily, many points were left unsettled; to be agreed upon at some future meeting of the Association, or determined by the individual colleges. And one of these points left unsettled is, is our opinion, of fundamental importance: *the amount of fees to be charged*. All the schools entering into the organization, having agreed to a three years course of six months each (a big advance, upon which the whole country is to be congratulated), all requiring the same standard for matriculation, it would be eminently right and proper and just that all should charge the same fee for tuition.

The profession has been prating for years of "elevating the standard of medical education;" but they have always begun at the wrong end; they have asked the legislature to make laws which will permit a board in each State to fix an arbitrary standard of requirement, and subject all new comers to examination, and have been furiously indignant because they were refused.

The JOURNAL has contended all along that we should begin at the fountain head; that the colleges should raise the standard of requirement both for matriculation and graduation, and it congratulates itself now on this beginning. True, it is far short of what it ought to be, but it is a beginning, and if the colleges do not find, like Bellevue did, that it cuts off too much revenue, and bolt the organization, we may hope that in time a diploma will come to mean more than the average diploma means now,—that the owner has paid one or two $50 fees, and gone through the form of one or two six weeks courses of lectures. So mote it be.

OFFICERS OF THE ASSOCIATION OF SOUTHENN MEDICAL COLLEGES.—Prof. J. M. Bodine, M. D., University of Louisville, President; Prof. W. D. Haggard, M. D., Medical Department University of Tennessee, Vice-President; Prof. G. C. Savage, Medical Department Vanderbilt University, Secretary and Treasurer.

The concurrence of two-thirds of the Southern Medical Schools is necessary to carry into effect the rules and regulations adopted; but that number were represented at the meeting, either in person or by letter, so it is settled, we suppose. We understand that the Medical Department of lhe University of Tennessee, and the Medical Department of the University of the South (Sewanee Medical College), the Knoxville Medical School and the St. Louis Medical School [?] had already adopted the three years course, previous to the meeting. The Texas Medical College (Medical Department University of Texas) had led off, we believe, with a three years course, graded.

